# Phase 2 multicenter maintenance study of golidocitinib, A JAK1 selective inhibitor, in patients with peripheral T cell lymphomas after first-line systemic therapy (JACKPOT26)

**DOI:** 10.1038/s41408-026-01452-8

**Published:** 2026-03-17

**Authors:** Juying Wei, Qingqing Cai, Liling Zhang, Liqun Zou, Zengjun Li, Keshu Zhou, Huijing Wu, Lihua Qiu, Liping Su, Kaiyang Ding, Hui Zhou, Li Yu, Fei Li, Wenyu Li, Li’e Lin, Qing Xiao, Erhua Wang, Hongmei Jing, Meifang Zheng, Hongyu Zhang, Yuhuan Gao, Da Gao, Lijia Chen, Jie Jin

**Affiliations:** 1 The First Affiliated Hospital, Zhejiang University School of Medicine, Hangzhou, China; 2 Sun Yat-sen University Cancer Center, Guangzhou, China; 3 Wuhan Union Hospital, Wuhan, China; 4 West China Hospital, Sichuan University, Chengdu, China; 5 Shandong Cancer Hospital, Jinan, China; 6 Henan Cancer Hospital, Zhengzhou, China; 7 Hubei Cancer Hospital, Wuhan, China; 8 Tianjin Medical University Cancer Institute & Hospital, Tianjin, China; 9 Shanxi Provincial Cancer Hospital, Taiyuan, China; 10 Anhui Provincial Cancer Hospital, Hefei, China; 11 Hunan Cancer Hospital, Changsha, China; 12 The Second Affiliated Hospital of Nanchang University, Nanchang, China; 13 The First Affiliated Hospital of Nanchang University, Nanchang, China; 14 Guangdong Provincial People’s Hospital, Guangzhou, China; 15 Hainan General Hospital, Haikou, China; 16 The First Affiliated Hospital of Chongqing Medical University, Chongqing, China; 17 The Third Xiangya Hospital, Central South University, Changsha, China; 18 Peking University Third Hospital, Beijing, China; 19 Linyi Cancer Hospital, Linyi, China; 20 The Fifth Affiliated Hospital, Sun Yat-sen University, Zhuhai, China; 21 The Fourth Hospital of Hebei Medical University and Hebei Tumor Hospital, Shijiazhuang, China; 22 The Affiliated Hospital of Inner Mongolia Medical University, Hohhot, China; 23 Dizal Pharmaceutical, Shanghai, China

**Keywords:** Molecularly targeted therapy, Drug development, Translational research, Phase II trials, T-cell lymphoma

## Abstract

Patients with peripheral T cell lymphoma (PTCL) who achieved tumor response with first-line standard therapy were at high risk of disease relapse. We explored golidocitinib (150 mg once daily) as maintenance therapy for this group of patients (JACKPOT26, NCT06511869). This study included two cohorts: patients achieving a complete response (Cohort 1 (CR), *N* = 30) and a partial response (Cohort 2 (PR), *N* = 18) during induction stage. All enrolled patients were transplant ineligible or did not have a transplant plan. All dosed patients were included in the efficacy and safety analysis. In Cohort 1, the 24-month disease free survival (DFS) rate was 74.2% with golidocitinib treatment. In nodal subtypes (AITL, NOS, ALK- ALCL), the 24-month DFS rate was 62.7%. In Cohort 2, median progression free survival (PFS) was 17.4 months, and 24-month PFS rate was 48.6%. Nine out of 18 patients with initial PR achieved complete response, leading to a complete response rate of 50.0%, and median duration of response of 23.9 months. The most common ≥grade 3 treatment-related treatment-emergent adverse events (TRAEs) were hematological adverse events in nature, including neutrophil count decreased (47.9%), white blood cell count decreased (31.3%), lymphocyte count decreased (14.6%) and leukopenia (12.5%). The majority of these TRAEs were reversible and clinically manageable. TRAEs leading to treatment interruption and discontinuation occurred in 60.4% and 10% of patients, respectively. No TRAEs leading to fatal outcomes were reported. This study suggests the potential of golidocitinib as maintenance therapy for patients with PTCL.

## Introduction

Peripheral T-cell lymphoma (PTCL) is a highly heterogenous and aggressive non-Hodgkin lymphoma (NHL), which represent approximately 5%-10% and 15%-20% of NHLs in Western and Asian countries, respectively [1]. The standard first-line therapies included brentuximab vedotin combined with cyclophosphamide, doxorubicin and prednisone (BV + CHP) for CD30+ anaplastic large cell lymphomas (ALCL), dexamethasone, cisplatin, gemcitabline and pegaspargase (DDGP) for natural killer/T-cell lymphoma (NK/TCL), and cyclophosphamide, doxorubicin, vincristine, prednisone (CHOP) for the rest subtypes [2, 3]. The occurrence of disease relapse in PTCL is highly prevalent after the initial treatment. The estimated 2-year disease free survival (DFS) and progression-free survival (PFS) rates for patients who achieved complete response (CR) and partial response (PR) after the initial chemotherapy were 63.4% and 19.3%, respectively [4]. This suggests that 36% of patients with CR and 80% of patients with PR relapsed within two years after completion of first-line therapy. The prognosis of relapsed pateints was poor. The estimated 2-year overall survival (OS) rate for the CR patients was 75.3%, whereas median OS was 11-15.9 months for the patients who did not achieve CR after the first-line treatment [4, 5]. Currently, there was no consensus on maintenance therapy for patients who are ineligible for stem cell transplantation after achieving tumor reponse with first-line therapy. Therefore, there is an unmet medical need to develop therapeutical agents to maintain or ehance remission after first-line treatment of PTCL.

Mechanism of disease relapse for PTCL patients who achieved tumor reponse after first-line therapy may arise from a cancer ecosystem composed of multiple sources, including the inherent aggressiveness of the disease, the presence of minimal residual disease (MRD) for CR patients or residual tumor cells for PR patients after initial treatment, drug resistance to chemotherapy, heterogeneous cancer cells themselves, their surrounding immune related tumor microenvironment, and the signaling pathways of lymphoma cells [6]. Recent genetic analyses of PTCL have suggested that the activation mutations of nuclear factor-κB (NF-κB), Notch, janus kinase (JAK)/signal transducter and activator of transcription 3 (STAT3), ras homology A (RHOA) and phosphatidylinositol 3-kinase (PI3K)/AKT signaling pathways play an important role in the pathogenesis of PTCL [7]. Mutations in genes encoding proteins involved in epigenetic regulation are also seen in various malignancies including PTCL, particularly in those expressing follicular helper T cell (TFH) differentiation markers, such as angioimmunoblastic T-cell lymphoma (AITL) and some PTCL-not otherwise specified (NOS).

Constitutive activation of JAK/STAT pathways plays an important role in T-cell malignancies [8–11]. The expression of JAK/STAT pathway genes is upregulated in PTCL, and mutations in JAK3, STAT3, and STAT5B lead to constitutive activation of JAK/STAT pathway [7]. In particular, activation of STAT3 through phosphorylation at Y705 (pSTAT3) is reported in up to 70% of human tumors [12]. Among JAK family, JAK1 is considered to be the primary driver of STAT3 pathway activation. In contrast, targeting other JAK family members is associated with toxicities (e.g. JAK2 inhibition-related severe anemia), which limits clinical application [13]. Therefore, selective JAK1 inhibition could be a viable means to enable effective target coverage of JAK/STAT3 signaling pathway by sparing toxicities associated with hitting other JAK family members [14].

Golidocitinib (AZD4205) is an oral Janus kinase 1 (JAK1) selective tyrosine kinase inhibitor [15], which was granted conditional approval in China for the treatment of relapsed/refractory (r/r) PTCL based on the results of a phase 2 multinational pivotal study JACKPOT8 part B (NCT04105010). In its phase 2 study, golidocitinib showed remarkable and durable antitumor activity in patients with r/r PTCL, with manageable safety profile [16]. With its long-term durable tumor response in patients with r/r PTCL, golidocitinib was evaluated as maintenance therapy in patients who achieved tumor response after first-line standard therapy in a phase 2 multi-center clinical study (JACKPOT26, NCT06511869). Here we reported the results of this phase 2 study.

## Methods

### Study design and participants

This is a phase 2, open-label, single arm, multi-center trial of golidocitinib across 22 clinical centers in China. This study complies with the ethical principles of Good Clinical Practice (GCP) as required by the regulatory agencies, and in accordance with the Declaration of Helsinki. The study was approved by site ethics committee (EC) or Institutional Review Board (IRB) before initiation.

All patients provided written, informed consent before participation. Eligible patients (aged ≥18 years) had pathological diagnosis of PTCLs and Eastern Cooperative Oncology Group (ECOG) performance status (PS) of 0 to 1. Pathological diagnosis was performed by local laboratories for study entry. The PTCL subtypes included: PTCL-NOS, AITL, anaplastic lymphoma kinase-negative ALCL (ALK- ALCL), enteropathy-associated T-cell lymphoma (EATL), monomorphic epitheliotropic intestinal T-cell lymphoma (MEITL), NK/TCL, hepatosplenic T-cell lymphoma (HSTCL), and subcutaneous panniculitis like T-cell lymphoma (SPTCL), according to the 2016 revision of the World Health Organization (WHO) classification of lymphoid neoplasms.

Patients who achieved complete or partial response with first-line standard therapies were eligible for this study. The choice of first-line therapy was based on histological subtype of PTCL and local clinical practice. Transplant ineligible patients or patients without a transplant plan were enrolled. There were two cohorts in this study: Cohort 1 (complete response, CR) and Cohort 2 (partial response, PR). For patients who achieved CR (Cohort 1), the maximum duration between completion of first-line treatment and entry into this study was 3 months, under the condition that treatment-emergent adverse events (TEAEs) related to first-line therapies have been resolved to ≤ grade 1 according to Common Terminology Criteria for Adverse Events (CTCAE, version 5.0). In addition, patients must have adequate organ and system functions, e.g. hematological, hepatic and renal function parameters meeting the following criteria: absolute neutrophil count ≥1.5 × 10^9/L, platelet counts ≥ 100 × 10^9/L, hemoglobin ≥8 g/dL, total bilirubin ≤ 1.5 × upper limit of normal (ULN), aspartate aminotransferase (AST) and alanine aminotransferase (ALT) ≤ 2.5 × ULN, and creatinine ≤1.5 × ULN), to participate into the study. Patients in Cohort 2 were also required to meet the above safety requirements. In addition, they were not required to have measurable tumor lesions at baseline. Totally 48 patients were enrolled, with 30 and 18 patients in Cohort 1 and Cohort 2, respectively.

### Procedures

Golidocitinib was administered orally at 150 mg once daily (QD) until disease relapse/progression, unacceptable toxicities, the maximum treatment duration reached, use of new anti-cancer therapy, withdrawal of informed consent, death, or termination of the study by sponsor (whichever came first). For Cohort 1 (CR), the maximum treatment duration was 12 months. For Cohort 2 (PR), continuous dosing was required until a satisfactory tumor response was achieved and sustained (lasting for more than 1 year), and the maximum treatment duration did not exceed 2 years.

Tumor response was assessed by investigators per Lugano 2014 criteria, based on computed tomography (CT) scans. For Cohort 1, it was conducted from 12 weeks since the first dose until disease relapse or completion of 2-year follow-up (Day 1 of Cycle 4, 7, 10, and 14, then every 4 months thereafter). For Cohort 2, it was conducted from 8 weeks since the first dose until disease progression (Day 1 of Cycle 3, 5, 7, 10, and 13, then every 3 cycles thereafter).

Adverse events were assessed by investigators during the study period, coded per Medical Dictionary for Regulatory Activities Terminology (MedDRA, version 28.0) and graded according to the CTCAE (version 5.0). Treatment interruption and/or dose reduction were applied to manage adverse events.

### Outcomes

The primary endpoint included adverse events (AEs) and serious adverse events (SAEs) for both cohorts. The secondary endpoints included 12-month DFS rate and DFS for Cohort 1; objective response rate (ORR), duration of response (DoR), 12-month PFS rate and PFS for Cohort 2. The other endpoints included 24-month DFS rate for Cohort 1, 24-month PFS rate for Cohort 2, and OS for both cohorts. DFS was defined as the time from the first dose until disease relapse or death, whichever occurred first. The 12-month and 24-month DFS rates were defined as the rates of patients remaining disease free at the specified time points. PFS was defined as the time from the first dose until disease progression or death, whichever occurred first. The 12-month and 24-month PFS rates were defined as the rates of patients remaining progression free at the specified time points. ORR was defined as the proportion of patients achieving CR or PR. DoR was defined as the time from the first CR or PR until disease progression or death, whichever occurred first. OS was defined as the time from the first dose until death from any cause. Relative dose intensity was defined as the ratio of the actual dose intensity to the planned dose intensity as prescribed in the protocol. The formula used was actual dose intensity/150 × 100%, where actual dose intensity was calculated as the cumulative dose divided by the number of days between the first and last dose (inclusive), with treatment interruptions occurring after the last dose not being accounted for in the calculation.

### Statistical analysis

The continuous variables were summarized using descriptive statistics, and the number and percentage were provided for categorical variables. The time to event endpoints and their rates were analyzed using Kaplan-Meier method. The 95% confidence interval (CI) for ORR was estimated based on the exact method. All treated patients were included in the patient demographics and baseline characteristics, efficacy and safety analysis. Additionally, DFS and PFS were analyzed across different PTCL subtypes (NOS, AITL, ALK-ALCL, NK/TCL, and other), as well as by nodal versus non-nodal PTCL. Nodal PTCL included NOS, AITL and ALK- ALCL. DFS, PFS, and OS were also analyzed for patients receiving maintenance treatment within and beyond 1.5 months from the completion of frontline therapy, respectively. A separate analysis of ORR was performed in patients with baseline measurable tumor lesions of Cohort 2. All statistical analyses were performed using SAS (version 9.4).

## Results

### Patient demographics and baseline characteristics

**Fig. 1:**
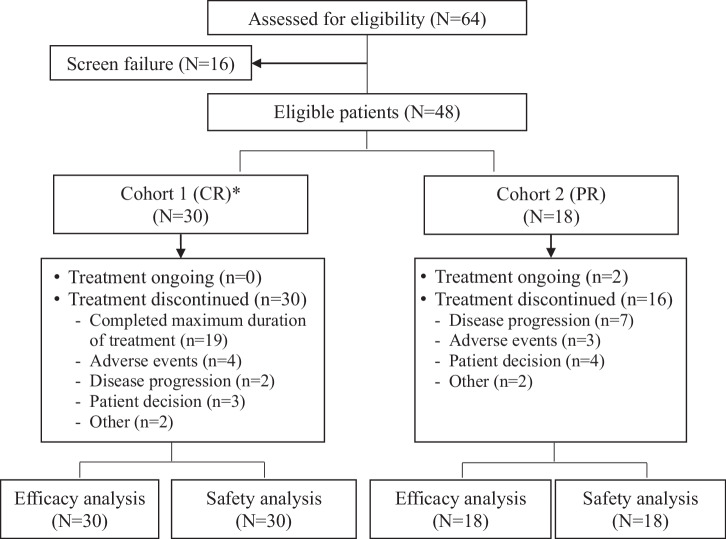
Trial profile (JACKPOT26). * In Cohort 1, the maximum duration on treatment was 12 months. CR: complete response; PR: partial response.

Between March 17, 2022 and March 16, 2023, a total of 48 patients who met eligibility criteria were enrolled, and among them, 30 and 18 patients were enrolled into Cohort 1 and Cohort 2, respectively (Fig. 1).

Patient demographics and baseline characteristics were shown in Table 1. All patients are Asian. The median age was 58.5 years (range: 25 - 74), and the majority were male (52.1%). 31.3% of patients had ECOG PS of 1. The major pathology subtypes included AITL (31.3%), NOS (27.1%) and NK/TCL (27.1%). At baseline, 8.3% of patients had elevated serum lactate dehydrogenase (LDH) levels. At initial diagnosis, 6.3% of patients had extranodal involvement and 8.3% of patients had combined nodal and extranodal involvement, most of patients (66.7%) presented with advanced disease (stage III or above). All patients have received standard systemic therapies according to different histological subtypes and clinical practice. For patients with NK/TCL, all of them received pegaspargase-containing therapy and the majority (61.5%) received p-GemOx chemotherapy with or without PD-1inhibitor. CHOP-based therapies were used in all patients with PTCL nodal type or other types. Etoposide, chidamide (an histone deacetylase (HDAC) inhinitor), azacitine, and thalidomine were most commonly added into CHOP therapy. Nine patients (18.8%) received prior radiotherapy. The median time from completion of frontline therapy to receive golidocitinib treatment was 1.7 months.

**Table 1:** Patient demographics and baseline characteristics.

	Cohort 1 (*N* = 30)	Cohort 2 (*N* = 18)	Total (*N* = 48)
Age (years)			
Median (min, max)	56.5 (25, 70)	61.5 (31, 74)	58.5 (25, 74)
Gender, *n* (%)			
Male/Female	16 (53.3)/14 (46.7)	9 (50.0)/9 (50.0)	25 (52.1)/23 (47.9)
Baseline ECOG PS, *n* (%)			
0/1	22 (73.3)/8 (26.7)	11 (61.1)/7 (38.9)	33 (68.8)/15 (31.3)
Histopathological Subtypes, *n* (%)			
PTCL-not otherwise specified (PTCL, NOS)	6 (20.0)	7 (38.9)	13 (27.1)
Angioimmunoblastic T-cell lymphoma (AITL)	8 (26.7)	7 (38.9)	15 (31.3)
Anaplastic large cell lymphoma (ALCL)	3 (10.0)	0 (0.0)	3 (6.3)
Natural killer/T-cell lymphoma (NK/TCL)	10 (33.3)	3 (16.7)	13 (27.1)
Other^a^	3 (10.0)	1 (5.6)	4 (8.3)
Serum LDH Elevation at the Baseline, *n* (%)			
Yes/No	2 (6.7)/28 (93.3)	2 (11.1)/16 (88.9)	4 (8.3)/44 (91.7)
Primary Tumor Type, *n* (%)			
Lymph node/lymph mass	28 (93.3)	13 (72.2)	41 (85.4)
Extra-nodal lesion	1 (3.3)	2 (11.1)	3 (6.3)
Both lymph node and Extra-nodal	1 (3.3)	3 (16.7)	4 (8.3)
Ann Arbor Staging at Initial Diagnosis, *n* (%)			
Stage I	3 (10.0)	0 (0.0)	3 (6.3)
Stage IE	2 (6.7)	0 (0.0)	2 (4.2)
Stage II	8 (26.7)	1 (5.6)	9 (18.8)
Stage IIE	2 (6.7)	0 (0.0)	2 (4.2)
Stage III	7 (23.3)	8 (44.4)	15 (31.3)
Stage IV	8 (26.7)	9 (50.0)	17 (35.4)
Types of Prior Anti-cancer Therapies by Subtype, *n* (%)			
Nodal	17	14	31
CHOP-based^b^	17 (100.0)	14 (100.0)	31 (100.0)
NK/TCL	10	3	13
p-GEMOX	6 (60.0)	2 (66.7)	8 (61.5)
Other pegaspargase-containing therapies	4 (40.0)	1 (33.3)	5 (38.5)
Other^a^	3	1	4
CHOP-based^b^	3 (100.0)	1 (100.0)	4 (100.0)
Prior Radiotherapy, *n* (%)	7 (23.3)	2 (11.1)	9 (18.8)
Time from Completion of Frontline Therapy to First Dose of Golidocitinib (months)^§^			
Median (min, max)	1.8 (0, 4)	1.5 (0, 5)	1.7 (0, 5)

*ECOG* : Eastern Cooperative Oncology Group.

^a^Other included subcutaneous panniculitis like T-cell lymphoma (*n* = 2), hepatosplenic T-cell lymphoma (*n* = 1) and monomorphic epitheliotropic intestinal T-cell lymphoma (*n* = 1). ^§^Six patients in Cohort 1 started golidocitinib treatment >3 months from completion of first-line therapy. The investigators considered that, despite the interval exceeded 3 months, they could still benefit from this study given the poor prognosis of PTCL.

^b^CHOP-based regimens involved CHOP as the foundation, supplemented with other drugs (such as etoposide, chidamide (an HDAC inhibitor), azacitine, thalidomine, brentuximab vedotin, cisplatin, decitabine, and cytarabine).

### Efficacy

**Fig. 2:**
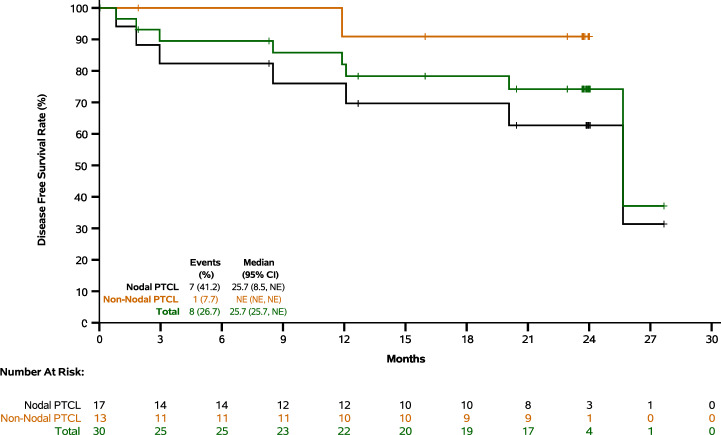
Disease free survival (Cohort 1). Nodal subtypes included NOS (PTCL, not otherwise specified), AITL (angioimmunoblastic T-cell lymphoma) and ALCL (anaplastic large cell lymphoma). Non-nodal subtypes included NK/TCL (natural killer/T-cell lymphoma), SPTCL (subcutaneous panniculitis-like T-cell lymphoma) and MEITL (monomorphic epitheliotropic intestinal T-cell lymphoma). NE: not evaluable.

In Cohort 1, with median follow-up of 23.8 months, 8 out of 30 patients (26.7%) had events of disease recurrence or death, with 12-month and 24-month DFS rates of 82.1% and 74.2%, respectively. In patients with nodal and non-nodal subtypes, the 12-month DFS rates were 76.0% and 90.9%, and 24-month DFS rates were 62.7% and 90.9%, respectively (Fig. 2 and Supplementary Table S1). In patients with NOS, AITL and ALK- ALCL subtypes, the 12-month DFS rates were 66.7%, 75.0% and 100.0%, and 24-month DFS rates were 66.7%, 50.0% and not estimable (NE), respectively (Supplementary Fig. S1 and Supplementary Table S2). In patients receiving maintenance treatment within and beyond 1.5 months from the completion of frontline therapy, the 12-month DFS rates were 90.0% and 77.5%, and 24-month DFS rates were 90.0% and 65.1%, respectively (Supplementary Table S3).

**Fig. 3:**
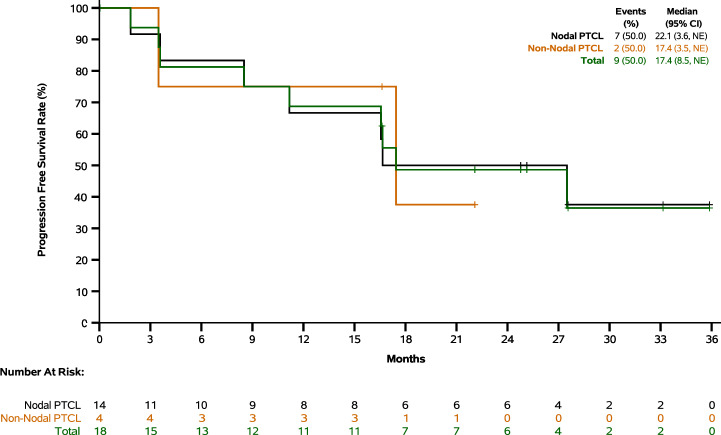
Progression free survival (Cohort 2). Nodal subtypes included NOS (PTCL, not otherwise specified) and AITL (angioimmunoblastic T-cell lymphoma). Non-nodal subtypes included NK/TCL (natural killer/T-cell lymphoma) and HSTCL (hepatosplenic T-cell lymphoma). NE: not evaluable.

**Fig. 4:**
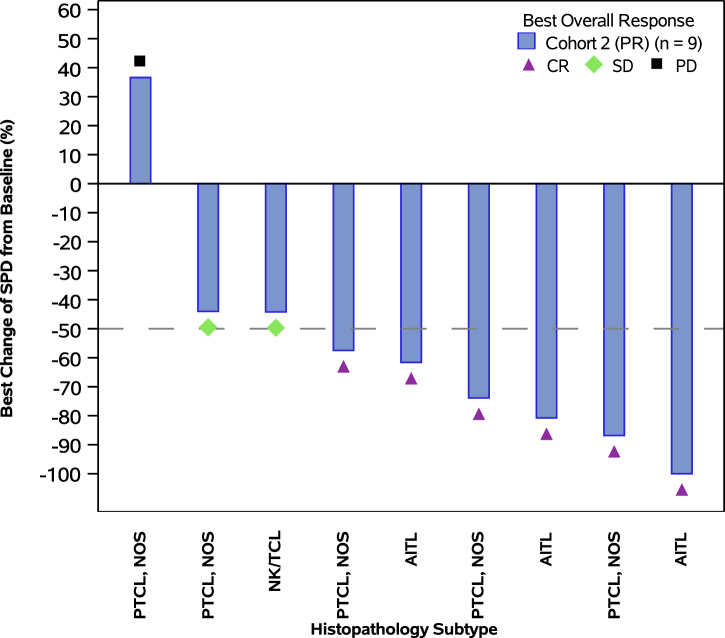
The best change of target lesions with golidocitinib treatment (Cohort 2). CR: complete response; PR: partial response; SD: stable disease.

In Cohort 2, with median follow-up of 25.1 months, the median PFS was 17.4 months, and the longest PFS reached 35.9 months. The 12-month and 24-month PFS rates were 68.8% and 48.6%, respectively. In patients with nodal and non-nodal subtypes, the median PFS were 22.1 and 17.4 months, respectively (Fig. 3 and Supplementary Table S4). In patients receiving maintenance treatment within and beyond 1.5 months from the completion of frontline therapy, the median PFS were 27.5 and 17.4 months, respectively (Supplementary Table S3). Among all 18 patients (regardless of baseline measurable tumor lesions), 9 achieved tumor response (all were complete response), with a complete response rate (CRR) of 50.0% and a median time to CR of 5.5 months (Table 2). Among 10 patients with baseline measurable tumor lesions, 8 had target lesion shrinkage and 6 achieved tumor response (all were complete response), with an CRR of 60.0% and a median time to CR of 5.5 months (Fig. 4 and Supplementary Table S5). The CRRs of NOS, AITL and others were 57.1%, 57.1%, and 25.0%, respectively. With median follow-up of 19.4 months, the median DoR was 23.9 months (Supplementary Table S6).

**Table 2:** Objective response rate (Cohort 2).

	Cohort 2 (*N* = 18)
Objective Response Rate, *n* (%)	9 (50.0)
95% CI^a^	(26.0, 74.0)
Best Overall Response, *n* (%)	
Complete Response	9 (50.0)
Partial Response	0 (0.0)
Stable Disease	6 (33.3)
Progressive Disease	1 (5.6)
Not Evaluable	2 (11.1)
Median Time to Complete Response (months)	5.5

^a^The 95% CI was estimated based on the exact (Clopper-Pearson) method.

With median follow-up of 30.5 and 28.6 months, the median OS have not been reached for both cohorts, and the 24-month OS rates were 89.5% and 75.0%, respectively (Supplementary Fig. S2 and Supplementary Table S7). In patients receiving maintenance treatment within and beyond 1.5 months from the completion of frontline therapy, the 24-month OS rates were 94.1% and 78.1%, respectively (Supplementary Table S3).

### Safety

In Cohort 1, the median duration of exposure was 11.9 months, and 63.3% of patients completed the planned 12-month treatment, with relative dose intensity of 94.0%. In Cohort 2, the median duration of exposure was 13.4 months, with relative dose intensity of 94.1%.

Among all 48 patients, 72.9% of patients experienced treatment-related adverse events (TRAEs) with grade ≥3, and 29.2% of patients experienced treatment-related serious adverse events (TRSAEs) (Supplementary Table S8). The most common TRAEs with grade ≥3 included neutrophil count decreased (47.9%), white blood cell count decreased (31.3%), lymphocyte count decreased (14.6%), pneumonia (14.6%) and leukopenia (12.5%). Although AST increased and ALT increased in all grades were reported in 43.8% and 33.3% of the patients, but only 2.1% and 0.0% was grade ≥3 in severity (Table 3).

**Table 3:** Common Treatment-related Adverse Events (>20%).

	Cohort 1 (*N* = 30) *n* (%)		Cohort 2 (*N* = 18) *n* (%)		Total (*N* = 48) *n* (%)	
Preferred Term	All Grades	Grade ≥ 3	All Grades	Grade ≥ 3	All Grades	Grade ≥ 3
Neutrophil count decreased	25 (83.3)	14 (46.7)	15 (83.3)	9 (50.0)	40 (83.3)	23 (47.9)
White blood cell count decreased	23 (76.7)	8 (26.7)	13 (72.2)	7 (38.9)	36 (75.0)	15 (31.3)
Platelet count decreased	20 (66.7)	1 (3.3)	11 (61.1)	2 (11.1)	31 (64.6)	3 (6.3)
Aspartate aminotransferase increased	12 (40.0)	0 (0.0)	9 (50.0)	1 (5.6)	21 (43.8)	1 (2.1)
Anaemia	13 (43.3)	1 (3.3)	4 (22.2)	0 (0.0)	17 (35.4)	1 (2.1)
Lymphocyte count decreased	12 (40.0)	4 (13.3)	4 (22.2)	3 (16.7)	16 (33.3)	7 (14.6)
Alanine aminotransferase increased	9 (30.0)	0 (0.0)	7 (38.9)	0 (0.0)	16 (33.3)	0 (0.0)
Blood creatine phosphokinase increased	6 (20.0)	0 (0.0)	6 (33.3)	1 (5.6)	12 (25.0)	1 (2.1)
Pneumonia	5 (16.7)	3 (10.0)	6 (33.3)	4 (22.2)	11 (22.9)	7 (14.6)
Leukopenia	6 (20.0)	4 (13.3)	5 (27.8)	2 (11.1)	11 (22.9)	6 (12.5)
Blood fibrinogen decreased	6 (20.0)	0 (0.0)	4 (22.2)	1 (5.6)	10 (20.8)	1 (2.1)
Blood creatinine increased	5 (16.7)	0 (0.0)	5 (27.8)	0 (0.0)	10 (20.8)	0 (0.0)
Hypercholesterolaemia	5 (16.7)	0 (0.0)	4 (22.2)	0 (0.0)	9 (18.8)	0 (0.0)
Hypertriglyceridaemia	5 (16.7)	1 (3.3)	4 (22.2)	1 (5.6)	9 (18.8)	2 (4.2)

The majority of TRAEs were recovered/recovering by the data cut-off date.

TRAE leading to treatment interruption, dose reduction and treatment discontinuation were reported in 60.4%, 16.7% and 10% of the patients. Neutrophil count decreased, platelet count decreased, and infection disease such as pneumonia, herpes zoster, were the most common (>10%) reasons for treatment interruption. And the most frequently reported TRAE which leads to dose reduction is white blood cell decrease, reported in 4.2% of patients. The TRAEs leading to treatment discontinuation were relatively scattered. Pneumonia and lymphocyte count decrease were reported but only occurred in 1 patient each (Supplementary Table S9).

No TRAEs leading to fatal outcome were reported. The majority of TRAEs were recovered/recovering.

## Discussion

This study enrolled patients with PTCL who achieved tumor response with first-line therapies. and were transplant ineligible or did not have a transplant plan. The distribution of subtypes was similar to what have been reported in the literature [17], with the most frequent subtypes of NOS, AITL and NK/TCL. Aligning with the subtypes, all patients received CHOP-based regimen as the first-line treatment for nodal subtypes, followed by 61.5% receiving p-GemOx regimen for NK/TCL. Given that all patients were responding to first-line treatment, the general condition of these patients were better than patients with r/r PTCL, including percentage of patients with ECOG of 1 and baseline LDH evaluation.

Golidocitinib showed an acceptable and manageable safety profile in PTCL patients as maintenance therapy after first-line treatment. The most common TRAEs with grade ≥3 included neutrophil count decreased, white blood cell count decreased, lymphocyte count decreased, pneumonia and leukopenia. Cytopenia were commonly observed in other JAK inhibitors studies but could be monitored and well managed in the clinic. Pneumonia was considered to be related to immunosuppression due to JAK/STAT pathway inhibition [18]. Drug related AST increased and ALT increased in all grades were reported in 43.8% and 33.3% patients, but only 1 AST increased case reported as grade ≥3 in severity. Overall, the safety profile was consistent with what we previously reported in r/r PTCL patients [16], and there was no emerging new safety risk was identified. But it seemed that a higher proportion of patients (72.9% vs 59%) experienced TRAEs with grade ≥3 compared to our previous report on r/r PTCL. This may be due to the longer duration of treatment in patients receiving maintenance therapy (median drug exposure of 11.9 months) than in patients with r/r PTCL. In this case, a relatively higher rate of TRAEs leading to treatment interruption (60.4% vs 38%) and dose reduction (16.7% vs 8%) were observed. But the treatment discontinuation rate was low and similar between these two studies, at 10% and 9%, respectively. From this perspective, it indicated that most TRAEs were manageable and recoverable, and treatment could continue.

Golidocitinib treatment demonstrated promising effect on maintaining and enhancing tumor response in patients with PTCL post first-line therapies. For patients in Cohort 1, median DFS was 25.7 months, and the 12-month and 24-month DFS rates were 82.1% and 74.2%, respectively. For patients in Cohort 2, the median PFS was 17.4 months, and the longest PFS reached 35.9 months. The 12-month and 24-month PFS rates were 68.8% and 48.6%, respectively. Nine out of 18 initial PR patients achieved CR after golidocitinib treatment, indicating PR patients may benefit from treatment to achieve better response, although the sample size was limited.

Based on our subgroup analysis, consistent efficacy was observed cross nodal or non-nodal subtypes. Subgroup analysis also suggested that patients receiving maintenance treatment within 1.5 months of completing chemotherapy experienced generally better DFS, PFS and OS than those who received subsequent treatment beyond 1.5 months. This finding underscore the importance of treatment timing in optimizing long-term benefit.

Some limitations of the present work should be noted. First, the trial design is single-arm and lacks a concurrent control group. Therefore, the observed efficacy and safety signals may be influenced by several biases which cannot be fully adjusted by statistical techniques. Second, the sample size remains modest, although adequate for an early-phase study. Third, the relative dose intensity is calculated without accounting for treatment interruptions that occur after the last dose, which does not fully capture the relatively high rates of treatment interruptions experienced. Fourth, local pathology diagnosis and 2016 revised WHO criteria were applied in this study. PTCLs are a group of heterogenous diseases with no standardized biomarker panels for the diagnosis and subtype classification. Lack of central pathology review may result in an inability to mitigate the risk of diagnostic discordance among local hematopathologists. Besides, a new nodal T-follicular helper (TFH) cell lymphoma subset was introduced into the 2022 revised WHO criteria which may have changed subtype analysis results reported in this study. Finally, when conducting qualitative assessments of non-target lesions, variability among different readers inevitably introduces subjectivity. As a result, true antitumor activity may be discounted, as some patients with true but non-measurable lesion shrinkage may be classified as having stable disease. Therefore, the generalizability of our findings to a broader, molecularly heterogeneous population remains tentative. Future trials should include centralized imaging assessments, use standardized definitions to define non-targeted progression, and, when feasible, require at least one measurable lesion at study entry.

Despite these limitations, the study provides the first prospective evidence that golidocitinib showed acceptable toxicity as maintenance therapy and contributed to achieve deep and sustained remissions in patients who achieved tumor response after first-line therapy. Taken together, further studies are warranted to confirm golidocitinib as potential maintenance therapy for patients with PTCL.

## Supplementary information

Supplementary Figures Tables

## Data Availability

Qualified researchers may request access to study documents (including the clinical study report, study protocol with any amendments, blank case report form, and statistical analysis plan) that support the methods and findings reported in this manuscript. Requested should be submitted to https://vivli.org/.
