# Supplementary material for: Phase 2 multicenter maintenance study of golidocitinib, A JAK1 selective inhibitor, in patients with peripheral T cell lymphomas after first-line systemic therapy (JACKPOT26)
Source: Blood Cancer J. 2026 Mar 17;16(1):36. doi: 10.1038/s41408-026-01452-8 (PMC13005011; doi:10.1038/s41408-026-01452-8)
Supplement: Supplementary file 1 — Supplementary Figures Tables [file 41408_2026_1452_MOESM1_ESM.pdf]

Supplementary Table S1. Disease free survival (Cohort 1)

	**Nodal (N = 17)**	**Non-Nodal (N = 13)**	**Total (N = 30)**
Disease Free Survival (months)			
Median (95% CI) [a]	25.7 (8.5, NE)	NE (NE, NE)	25.7 (25.7, NE)
Min, Max [b]	0.8, 27.7+	0.0+, 24.0+	0.0+, 27.7+
Disease Free Survival Rate (%) (95% CI) [a] at			
6 months	82.4 (54.7, 93.9)	100.0 (100.0, 100.0)	89.5 (70.9, 96.5)
12 months	76.0 (48.0, 90.3)	90.9 (50.8, 98.7)	82.1 (62.1, 92.1)
18 months	69.7 (41.7, 86.1)	90.9 (50.8, 98.7)	78.3 (57.9, 89.7)
24 months	62.7 (34.8, 81.4)	90.9 (50.8, 98.7)	74.2 (53.2, 86.8)
Follow-up Time (months)			
Median (95% CI) [c]	23.9 (20.4, 24.0)	23.7 (16.0, 24.0)	23.8 (22.9, 24.0)

[a] Estimated by Kaplan-Meier method. [b] Statistics were calculated regardless of event or censor. [c] Median of follow-up time were estimated by the reverse Kaplan-Meier method. '+' denoted censored. Two patients discontinued golidocitinib treatment due to reaching the maximum cycle of administration defined by the study protocol and later they received the new anti-cancer therapy. Consequently, they were censored at the last tumor assessment before new anti-cancer therapy according to statistical analysis rule. CI: confidence interval; NE: not evaluable.

Supplementary Table S2. Disease free survival of different subtypes (Cohort 1)

	**NOS (N = 6)**	**AITL (N = 8)**	**ALK- ALCL (N = 3)**	**NK/TCL (N = 10)**	**Other (N = 3)**
Disease Free Survival (months)					
Median (95% CI) [a]	25.7 (0.8, 25.7)	NE (3.0, NE)	NE (NE, NE)	NE (11.9, NE)	NE (NE, NE)
Min, Max [b]	0.8, 25.7	3.0, 27.7+	12.7+, 23.9+	0.0+, 24.0+	16.0+, 24.0+
Disease Free Survival Rate (%) (95% CI) [a] at					
6 months	66.7 (19.5, 90.4)	87.5 (38.7, 98.1)	100.0 (100.0, 100.0)	100.0 (100.0, 100.0)	100.0 (100.0, 100.0)
12 months	66.7 (19.5, 90.4)	75.0 (31.5, 93.1)	100.0 (100.0, 100.0)	87.5 (38.7, 98.1)	100.0 (100.0, 100.0)
18 months	66.7 (19.5, 90.4)	62.5 (22.9, 86.1)	100.0 (100.0, 100.0)	87.5 (38.7, 98.1)	100.0 (100.0, 100.0)
24 months	66.7 (19.5, 90.4)	50.0 (15.2, 77.5)	NE (NE, NE)	NE (NE, NE)	100.0 (100.0, 100.0)
Follow-up Time (months)					
Median (95% CI) [c]	24.0 (8.3, NE)	24.0 (23.9, 27.7)	20.4 (12.7, 23.9)	23.7 (0.0, 23.8)	24.0 (16.0, 24.0)

[a] Estimated by Kaplan-Meier method. [b] Statistics were calculated regardless of event or censor. [c] Median of follow-up time were estimated by the reverse Kaplan-Meier method. '+' denoted censored. CI: confidence interval; NE: not evaluable.

Supplementary Table S3. Disease free survival, progression free survival and overall survival of patients receiving maintenance treatment within and beyond 1.5 months of completing frontline therapy

	**≤ 1.5 months**	**> 1.5 months**
**Disease Free Survival – Cohort 1**	**(N = 10)**	**(N = 20)**
Disease Free Survival (months)		
Median (95% CI) [a]	25.7 (3.0, 25.7)	NE (12.1, NE)
Min, Max [b]	3.0, 25.7	0.0+, 27.7+
Rate (%) (95% CI) [a] at		
6 months	90.0 (47.3, 98.5)	89.5 (64.1, 97.3)
12 months	90.0 (47.3, 98.5)	77.5 (50.5, 91.0)
18 months	90.0 (47.3, 98.5)	71.6 (44.4, 87.1)
24 months	90.0 (47.3, 98.5)	65.1 (37.9, 82.7)
Median Follow-up Time (95% CI) [c] (months)	23.9 (16.0, 24.0)	23.7 (12.7, 24.0)
**Progression Free Survival – Cohort 2**	**(N = 9)**	**(N = 9)**
Progression Free Survival (months)		
Median (95% CI) [a]	27.5 (3.6, 27.5)	17.4 (1.8, NE)
Min, Max [b]	0.0+, 27.5	1.8, 35.9+
Rate (%) (95% CI) [a] at		
6 months	85.7 (33.4, 97.9)	77.8 (36.5, 93.9)
12 months	71.4 (25.8, 92.0)	66.7 (28.2, 87.8)
18 months	57.1 (17.2, 83.7)	41.7 (10.9, 70.8)
24 months	57.1 (17.2, 83.7)	41.7 (10.9, 70.8)
Median Follow-up Time (95% CI) [c] (months)	24.8 (0.0, NE)	33.1 (16.6, 35.9)
**Overall Survival – Both Cohorts**	**(N = 19)**	**(N = 29)**
Overall Survival (months)		
Median (95% CI) [a]	NE (NE, NE)	NE (NE, NE)
Min, Max [b]	1.7+, 35.4+	0.8, 36.5+
Rate (%) (95% CI) [a] at		
6 months	100.0 (100.0, 100.0)	93.0 (74.7, 98.2)
12 months	100.0 (100.0, 100.0)	89.3 (70.2, 96.4)
18 months	94.1 (65.0, 99.1)	81.8 (61.7, 92.0)
24 months	94.1 (65.0, 99.1)	78.1 (57.6, 89.5)
Median Follow-up Time (95% CI) [c] (months)	30.5 (25.8, 31.9)	29.9 (28.3, 31.8)

[a] Estimated by Kaplan-Meier method. [b] Statistics were calculated regardless of event or censor. [c] Median of follow-up time were estimated by the reverse Kaplan-Meier method. '+' denoted censored. CI: confidence interval; NE: not evaluable.

Supplementary Table S4. Progression free survival (Cohort 2)

	**Nodal (N = 14)**	**Non-Nodal (N = 4)**	**Total (N = 18)**
Progression Free Survival (months)			
Median (95% CI) [a]	22.1 (3.6, NE)	17.4 (3.5, NE)	17.4 (8.5, NE)
Min, Max [b]	0.0+, 35.9+	3.5, 22.1+	0.0+, 35.9+
Progression Free Survival Rate (%) (95% CI) [a] at			
6 months	83.3 (48.2, 95.6)	75.0 (12.8, 96.1)	81.3 (52.5, 93.5)
12 months	66.7 (33.7, 86.0)	75.0 (12.8, 96.1)	68.8 (40.5, 85.6)
18 months	50.0 (20.8, 73.6)	37.5 (1.1, 80.8)	48.6 (22.9, 70.3)
24 months	50.0 (20.8, 73.6)	NE (NE, NE)	48.6 (22.9, 70.3)
Follow-up Time (months)			
Median (95% CI) [c]	27.6 (24.8, 35.9)	22.1 (16.6, 22.1)	25.1 (16.6, 33.1)

[a] Estimated by Kaplan-Meier method. [b] Statistics were calculated regardless of event or censor. [c] Median of follow-up time were estimated by the reverse Kaplan-Meier method. '+' denoted censored. One patient discontinued golidocitinib treatment due to an adverse event (lymphocyte count decreased) and later received the new anti-cancer therapy. Consequently, this patient was censored at the last tumor assessment before new anti-cancer therapy according to statistical analysis rule. CI: confidence interval; NE: not evaluable.

Supplementary Table S5. Objective response rate of patients with baseline measurable lesions (Cohort 2)

	**Cohort 2 (N = 10)**
Objective Response Rate, n (%)	6 (60.0)
95% CI [a]	(26.2, 87.8)
Best Overall Response, n (%)	
Complete Response	6 (60.0)
Partial Response	0 (0.0)
Stable Disease	2 (20.0)
Progressive Disease	1 (10.0)
Not Evaluable	1 (10.0)
Median Time to Complete Response (months)	5.5

[a] The 95% CI was estimated based on the exact (Clopper-Pearson) method. CI: confidence interval.

Supplementary Table S6. Duration of response (Cohort 2)

	**Cohort 2 (N = 18)**
Duration of Response (months)	
Median (95% CI) [a]	23.9 (9.3, NE)
Min, Max [b]	2.8+, 31.3+
Durable Response Rate (%) (95% CI) [a] at	
6 months	100.0 (100.0, 100.0)
12 months	71.4 (25.8, 92.0)
18 months	71.4 (25.8, 92.0)
24 months	47.6 (7.5, 80.8)
Follow-up Time (months)	
Median (95% CI) [c]	19.4 (2.8, 31.3)

[a] Estimated by Kaplan-Meier method. [b] Statistics were calculated regardless of event or censor. [c] Median of follow-up time were estimated by the reverse Kaplan-Meier method. '+' denoted censored. CI: confidence interval; NE: not evaluable.

Supplementary Table S7. Overall survival

	**Cohort 1 (N = 30)**	**Cohort 2 (N = 18)**
Overall Survival (months)		
Median (95% CI) [a]	NE (NE, NE)	NE (18.9, NE)
Min, Max [b]	0.8, 34.4+	1.7+, 36.5+
Survival Rate (%) (95% CI) [a] at		
6 months	96.7 (78.6, 99.5)	93.8 (63.2, 99.1)
12 months	96.7 (78.6, 99.5)	87.5 (58.6, 96.7)
18 months	89.5 (70.9, 96.5)	81.3 (52.5, 93.5)
24 months	89.5 (70.9, 96.5)	75.0 (46.3, 89.8)
Follow-up Time (months)		
Median (95% CI) [c]	30.5 (27.6, 31.8)	28.6 (24.9, 33.1)

[a] Estimated by Kaplan-Meier method. [b] Statistics were calculated regardless of event or censor. [c] Median of follow-up time were estimated by the reverse Kaplan-Meier method. '+' denoted censored. CI: confidence interval; NE: not evaluable.

Supplementary Table S8. Overview of TRAEs

	**Cohort 1 (N = 30) n (%)**	**Cohort 2 (N = 18) n (%)**	**Total (N = 48) n (%)**
Any Treatment-related TEAE	29 (96.7)	18 (100.0)	47 (97.9)
Any Treatment-related TEAE with Grade ≥3	20 (66.7)	15 (83.3)	35 (72.9)
Any Treatment-related Treatment-Emergent SAE	5 (16.7)	9 (50.0)	14 (29.2)
Any Treatment-related TEAE Leading to Treatment Interruption	17 (56.7)	12 (66.7)	29 (60.4)
Any Treatment-related TEAE Leading to Dose Reduction	4 (13.3)	4 (22.2)	8 (16.7)
Any Treatment-related TEAE Leading to Treatment Discontinuation	2 (6.7)	3 (16.7)	5 (10.4)
Any Treatment-related TEAE with Fatal Outcome	0 (0.0)	0 (0.0)	0 (0.0)

AEs with missing relationship were reported as related. SAE: serious adverse event; TEAE: treatment-emergent adverse event; TRAEs: treatment-related adverse events.

Supplementary Table S9. Common TRAEs leading to treatment adjustment

Preferred Term	Cohort 1 (N = 30) n (%)	Cohort 2 (N = 18) n (%)	Total (N = 48) n (%)
**Treatment Interruption (>10%)**			
Neutrophil count decreased	3 (10.0)	2 (11.1)	5 (10.4)
Platelet count decreased	3 (10.0)	2 (11.1)	5 (10.4)
Blood creatine phosphokinase increased	1 (3.3)	2 (11.1)	3 (6.3)
Pneumonia	5 (16.7)	5 (27.8)	10 (20.8)
Herpes zoster	3 (10.0)	2 (11.1)	5 (10.4)
Upper respiratory tract infection	2 (6.7)	2 (11.1)	4 (8.3)
**Dose Reduction**			
White blood cell count decreased	0 (0.0)	2 (11.1)	2 (4.2)
Blood fibrinogen decreased	1 (3.3)	0 (0.0)	1 (2.1)
Neutrophil count decreased	0 (0.0)	1 (5.6)	1 (2.1)
Platelet count decreased	0 (0.0)	1 (5.6)	1 (2.1)
Haemorrhage subcutaneous	1 (3.3)	0 (0.0)	1 (2.1)
Pneumocystis jirovecii pneumonia	1 (3.3)	0 (0.0)	1 (2.1)
Skin ulcer	0 (0.0)	1 (5.6)	1 (2.1)
Cardiac dysfunction	1 (3.3)	0 (0.0)	1 (2.1)
**Treatment Discontinuation**			
Hepatitis B reactivation	1 (3.3)	0 (0.0)	1 (2.1)
Herpes zoster	1 (3.3)	0 (0.0)	1 (2.1)
Pneumonia	0 (0.0)	1 (5.6)	1 (2.1)
Retinal disorder	0 (0.0)	1 (5.6)	1 (2.1)
Lymphocyte count decreased	0 (0.0)	1 (5.6)	1 (2.1)

TRAEs: treatment-related adverse events.

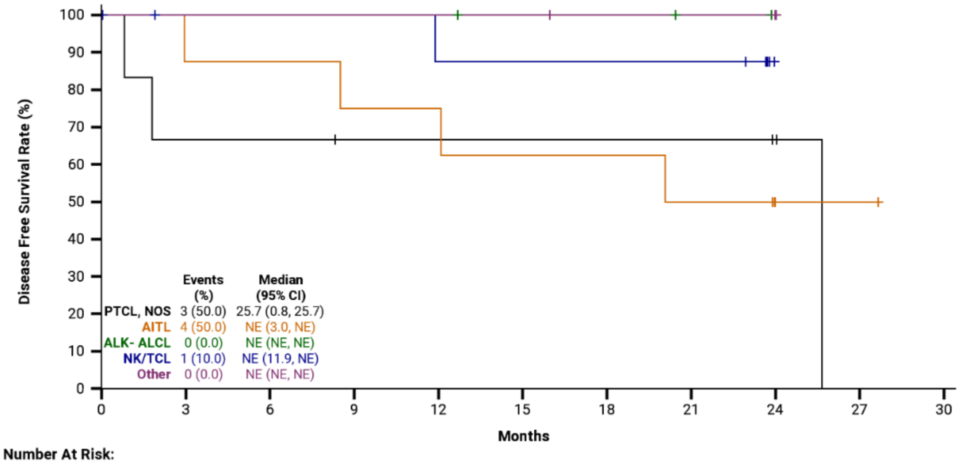

Number At Risk:

PTCL, NOS	6	4	4	3	3	3	3	3	2	0	0
AITL	8	7	7	6	6	5	5	4	1	1	0
ALK- ALCL	3	3	3	3	3	2	2	1	0	0	0
NK/TCL	10	8	8	8	7	7	7	7	0	0	0
Other	3	3	3	3	3	3	2	2	1	0	0

Supplementary Figure S1. Disease free survival of different subtypes (Cohort 1). Other subtypes included SPTCL (subcutaneous panniculitis-like T-cell lymphoma) and MEITL (monomorphic epitheliotropic intestinal T-cell lymphoma). NE: not evaluable.

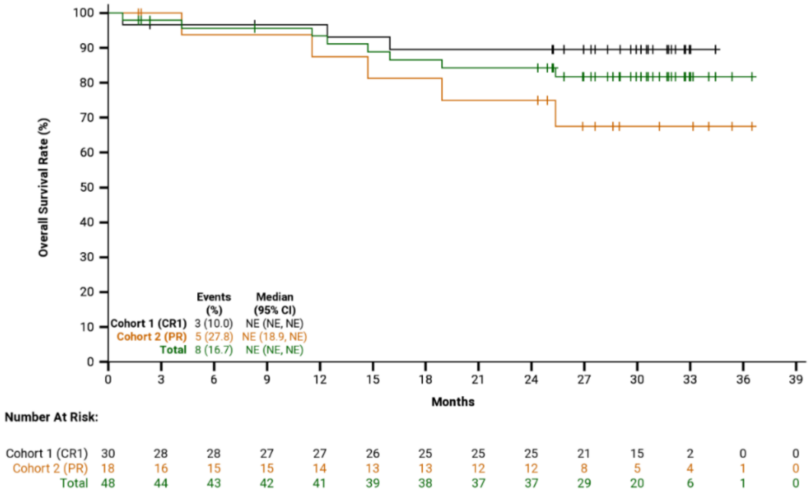

Supplementary Figure S2. Overall survival with golidocitinib treatment. NE: not evaluable.
